# Learning Contrast-Invariant Cancellation of Redundant Signals in Neural Systems

**DOI:** 10.1371/journal.pcbi.1003180

**Published:** 2013-09-12

**Authors:** Jorge F. Mejias, Gary Marsat, Kieran Bol, Leonard Maler, André Longtin

**Affiliations:** 1 Department of Physics, University of Ottawa, Ottawa, Ontario, Canada; 2 Department of Cellular and Molecular Medicine, University of Ottawa, Ottawa, Ontario, Canada; 3 Department of Biology, University of West Virginia, Morgantown, West Virginia, United States of America; 4 Centre for Neural Dynamics, University of Ottawa, Ottawa, Ontario, Canada; École Normale Supérieure, College de France, CNRS, France

## Abstract

Cancellation of redundant information is a highly desirable feature of sensory systems, since it would potentially lead to a more efficient detection of novel information. However, biologically plausible mechanisms responsible for such selective cancellation, and especially those robust to realistic variations in the intensity of the redundant signals, are mostly unknown. In this work, we study, via *in vivo* experimental recordings and computational models, the behavior of a cerebellar-like circuit in the weakly electric fish which is known to perform cancellation of redundant stimuli. We experimentally observe contrast invariance in the cancellation of spatially and temporally redundant stimuli in such a system. Our model, which incorporates heterogeneously-delayed feedback, bursting dynamics and burst-induced STDP, is in agreement with our *in vivo* observations. In addition, the model gives insight on the activity of granule cells and parallel fibers involved in the feedback pathway, and provides a strong prediction on the parallel fiber potentiation time scale. Finally, our model predicts the existence of an optimal learning contrast around 15% contrast levels, which are commonly experienced by interacting fish.

## Author Summary

The ability to cancel redundant information is an important feature of many sensory systems. Cancellation mechanisms in neural systems, however, are not well understood, especially when considering realistic conditions such as signals with different intensities. In this work, we study, employing experimental recordings and computational models, a cerebellar-like circuit in the brain of the weakly electric fish which is able to perform such a cancellation. We observe that *in vivo* recorded neurons in this circuit display a contrast-invariant cancellation of redundant stimuli. We employ a mathematical model to explain this phenomenon, and also to gain insight into several dynamics of the circuit which have not been experimentally measured to date. Interestingly, our model predicts that time-averaged contrast levels of around 15%, which are commonly experienced by interacting fish, would shape the circuit to behave as observed experimentally.

## Introduction

For many neural systems, prediction and cancellation of redundant signals constitutes one of the most convenient features for efficiently processing behaviorally meaningful information. When processing sensory input, for instance, neural circuits must be able to discriminate a novel stimulus from the background of redundant or non-relevant signals. A well-known situation in which such a discrimination may be highly advantageous is the so called “cocktail party problem”, in which a particularly relevant signal is extracted from a mixture containing other unimportant signals [1], [2]. This is known to be useful, for instance, to identify particular voices or sounds for both human and nonhuman animals [1], [3], or find and identify mates among conspecifics and heterospecifics [4]. However, the concrete mechanisms that the brain may employ to discriminate and cancel redundant information are presently unknown. It would be, therefore, convenient to identify and closely study natural systems displaying such a cancellation phenomenon, in order to isolate its fundamental principles. Of special interest might be the mechanisms able to conduct the cancellation process over a wide range of realistic conditions, such as canceling redundant signals of different intensities (or with time varying intensities due, e.g., to the relative movement of the receiver and the signal sources) while keeping novel stimuli intact.

The understanding of such a contrast-invariant cancellation mechanism would be beneficial not only for the “cocktail-party problem” in auditory systems, but also for visual neuroscience. Indeed, contrast invariance is a well known and well studied feature of the visual cortex, and particularly of the V1 area [5], [6]. A number of ingredients are thought to play a role in contrast invariance in V1, such as inhibition [7], [8], gain control [9], [10] or membrane fluctuations [9], [11], to name a few. However, many of the strategies giving rise to contrast invariance in V1 are still highly debated [12], [13] or simply starting to be uncovered [9], [14]. Consequently, new findings about how contrast invariance is achieved in other sensory modalities such as the simpler electrosensory system might contribute to understand contrast invariance in V1 and possibly to identify common principles for the corresponding biophysical mechanisms. The contrast-invariant cancellation sketched above stands as an interesting potential example.

A system able to perform cancellation of redundant information is known to exist in the electrosensory lateral-line lobe (ELL) of the weakly electric fish *Apteronotus leptorhynchus* [15]–[17]. This fish continuously emits a wave-type, high frequency (600∼100*Hz*) sinusoidal electric organ discharge (EOD) to sense its surroundings and communicate with conspecifics. Small objects such as prey produce spatially localized amplitude modulations (AMs) in the EOD. On the other hand, the presence of conspecifics or own-body movements such as tail bending induce spatially global AMs in the EOD. For example, since each fish has a fixed EOD frequency, the proximity of two fish produces an AM in the form of a beat of fixed frequency but time-varying amplitude due to the relative motion of the animals. The depth of these AMs, referred commonly as *contrast*, may depend on physical quantities such as the distance to conspecifics or the amplitude of the tail movement, in the case of global signals, or the size of (or distance to) the prey, in the case of local signals. Both spatially local and global AMs are encoded by electroreceptors (mainly, P-units) that densely cover the body of the fish [18]. In particular, AMs in the EOD are reliably encoded with a modulation in the firing rate of the P-units, which provide feedforward input to pyramidal neurons in the ELL. Interestingly, it has been found that a subpopulation of these pyramidal neurons, called superficial pyramidal (SP) cells, are able to respond selectively to local stimuli (i.e. prey) by removing low-frequency global redundant signals (i.e. tail bending), and thus maximizing the response to novel local stimuli [15], [19]. In the following, we will denote this pathway from the P-units to the SP cells as the *feedforward* pathway.

This removal of global signals is also present in another family of electric fish, namely the mormyrid weakly electric fish, although the mechanism differs significantly [20], [21]. These fish emit a pulse-type electric field instead of a wave-type field. The pacemaker generating the EOD also conveys spike discharges internally to ganglion neurons, to which the electroreceptors project. Through the so called anti-Hebbian spike-time-dependent plasticity, these ganglion neurons use this internal timing information (corollary discharge) to cancel out the redundant responses from the electroreceptors caused by the fish's own pulses, thus allowing an efficient detection of novel stimuli [22]. For both pulse-type and wave-type fish, the cancellation of global signals is achieved via the activation of a neural circuit denoted, by convention, as the indirect *feedback* pathway (it should be noted, however, that it is actually a longer feedforward circuit from the P-units to the SP cells via DP cells, as we will see below). Such a circuit, which we will denote here simply as feedback pathway, involves a granule cell population, the eminential granularis posterior (EGp), which projects a massive number of parallel fibers (PFs) onto SP cells.

In spite of this common architecture, the cancellation mechanism for the wave-type fish *A. leptorhynchus* is significantly different from the one used by pulse-type fish, not only because of the nature of its EOD (wave-type), but also because the corollary discharge is not present in wave-type fish. For the particular case of wave-type fish, the presence of burst-induced long-term plasticity in the PF-SP cell synapses [23], together with the segregation of the PFs into frequency-specific channels [16], [24], shapes the feedback input to the SP cell into a *negative image* of the redundant sensory stimulus, causing destructive interference and effectively canceling the global stimulus in the SP cells [16].

Little is known, however, about how different stimulus contrasts are processed in such a circuit. The AM contrast level of a signal is strongly correlated with the spatial proximity of the source, either for local input (i.e. distance to the prey) or global input (i.e. distance to conspecifics) [25], and thus constitutes a highly variable feature of the stimulus. Ideally, the fish would be expected to detect the presence of prey (and properly estimate the corresponding distance) while in the presence of other conspecifics at different distances from them. This would imply that SP cells display some form of cancellation for global stimuli of different contrasts. Neither the existence of such a cancellation nor its concrete dependence with the stimulus contrast have been experimentally quantified to date. Furthermore, the mechanisms that might lead to this phenomenon are not known. Arguably, a linear system would be expected to maintain the output as a given fraction of the input, regardless of the input strength. The neural circuits of interest here, however, are known to involve nonlinearities, including not only the input-output nonlinearity arising from the spiking threshold, but also the ones due to the presence of bursting and spike-timing dependent plasticity rules. Due to these nonlinearities, the particular configuration of PFs needed to properly cancel signals with a given contrast might be unable to provide consistent cancellation for another contrast. Also, since PFs are already segregated into different frequency-specific channels [16], it is unlikely that this strategy could be followed again to form contrast-specific channels, due to the limited number of PFs available. Therefore, both novel experimental observations and models are needed to address the question of cancellation of global stimuli with different contrasts.

In this work, we tackled this problem by employing a combination of experimental and modeling methods. We performed *in vivo* extracellular measurements of SP neuron activity for global and local stimuli of different AM frequencies and contrasts. Our measurements clearly show the existence of contrast-invariant cancellation of global stimulus for a wide, behaviorally relevant range of stimulus contrasts. Although a slight decay in cancellation for increasing contrasts is observed, the cancellation level decays only about 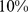 across all the range of contrasts considered, and thus cancellation remains at all times at values over 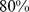 (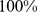 being a perfect cancellation of the global signal). In addition, a computational model is fitted to our *in vivo* data and the *in vitro* results presented in [23], and it is employed to explore the origins of this contrast invariance. Our model is based on those from previous studies [16], [24], which considered a feedback pathway composed of multiple delayed PFs projecting onto SP cells, with the strength of these PFs determined by a burst-induced long-term plasticity rule. In the model presented here, we also consider several novel mechanisms needed to understand contrast-invariant cancellation, which are: (*a*) the explicit modeling of the P-unit input/output characteristics, which affect both the feedforward and feedback pathways, and (*b*) the presence of saturating effects in the feedback pathway. We have also considered that plasticity does not shape PFs quickly, so that the model will have to deal with contrast levels that it was not explicitly trained to cancel. This last point is extremely important, as a system in which PFs are allowed, via long-term plasticity, to relearn how to cancel every new stimulus would be highly unrealistic.

Employing this highly detailed model fitted to our *in vivo* data, we find that (*i*) in spite of nonlinearities associated with PF plasticity, the level of AM contrast is successfully transmitted through the feedback pathway, matching the contrast arriving at SP cells through the feedforward pathway and explaining the contrast-invariant cancellation found *in vivo*, (*ii*) the PF weights associated with a given contrast are able to provide good cancellation for other contrast levels, and (*iii*) the minor decay of cancellation with contrast is due to the saturation of activity in the feedback pathway. In addition, our model predicts that, in order to properly cancel global signals at the experimentally observed levels, the average contrast level that must drive the PF learning lies around 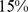 contrast levels. Interestingly, contrast levels around this one are commonly found within the natural environment for communication signals in the weakly electric fish [25]. This hypothetical link between social interaction and redundancy reduction in neural circuits might be used to uncover neural or synaptic mechanisms which are elusive to standard *in vivo* or *in vitro* techniques.

## Results

### Experimental observations

**Figure 1:**
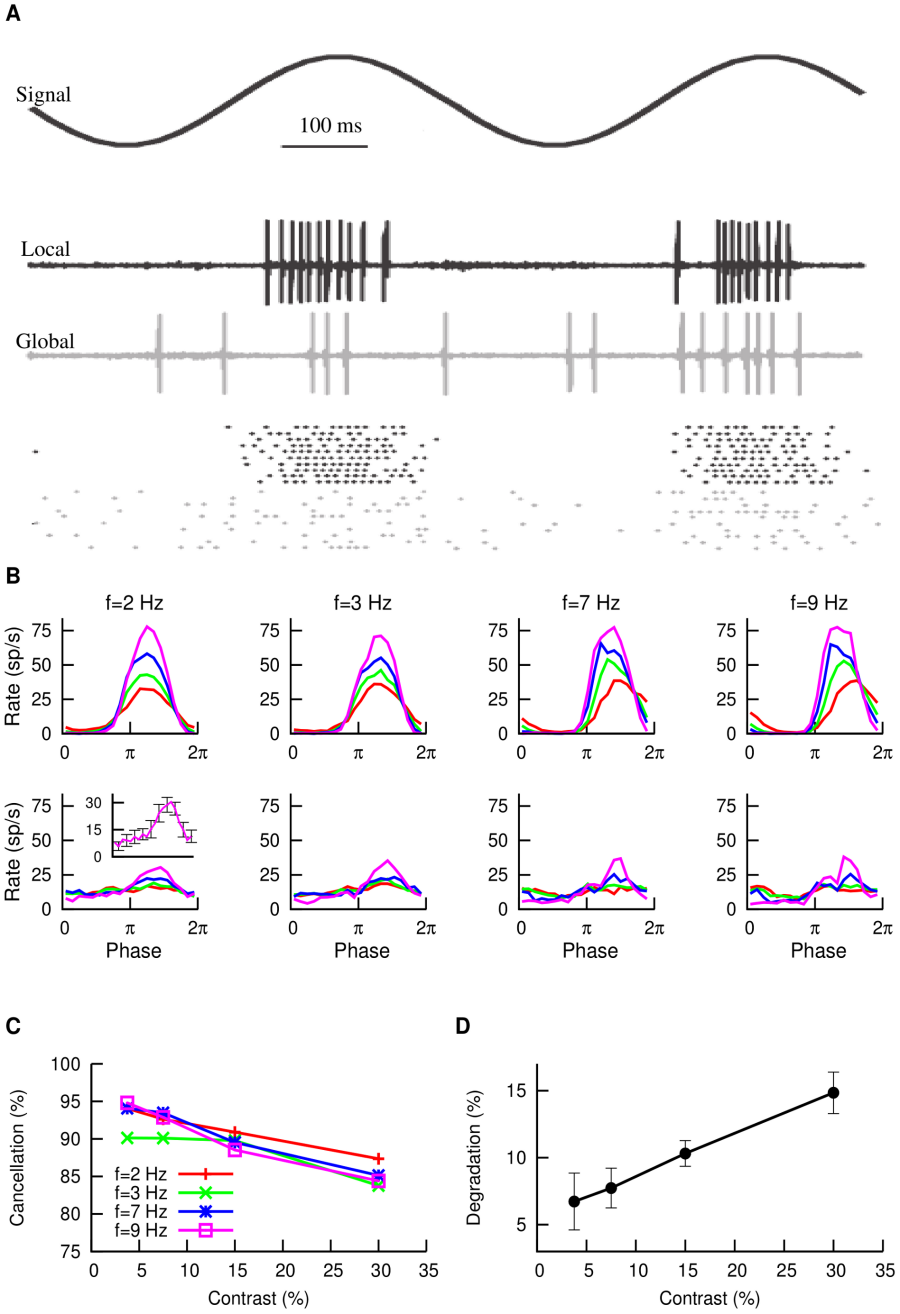
*In vivo* electrophysiological observations. (A) Response of SP cells to AM stimuli, for the case in which the signal is local (black) or global (gray). For each case, a single extracellular recording trial and a raster plot are shown. (B) Cancellation of global stimuli for different AM frequencies. Upper row corresponds to local stimulation while lower row corresponds to global stimulation. In each panel, the mean PSTH (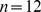 neurons from several fish) for contrasts of 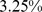 (red), 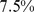 (green), 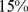 (blue) and 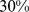 (violet) is displayed. The inset in the lower left panel shows the extent of the typical error bars for one of these curves (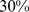). (C) Percentage of cancellation as a function of input contrast, for different AM frequencies. (D) Degradation of the signal cancellation, averaged over all AM frequencies considered, as a function of input contrast.

The goal of this study is to understand the mechanism that neurons in the ELL of the weakly electric fish employ to cancel spatially redundant signals with different contrasts. To do that, we first analyze experimental data from *in vivo* recordings. Fig. 1 shows the extracellularly recorded response of superficial pyramidal (SP) neurons in the ELL under different stimuli. As one can see, SP cells respond strongly and in a phase-locked fashion to local stimuli (Fig. 1A, black), whereas the response is much broader in phase, and smaller in amplitude, when the stimulus is global (Fig. 1A, gray). By considering peri-stimulus time histograms (PSTH), we confirm that, within the range of frequencies of the AM considered, the response to global signals is effectively cancelled (Fig. 1B). As previous studies have addressed, the cancellation is most pronounced within this AM frequency range [16], and it is achieved by the emergence of a *negative image* of the original signal, generated by the feedback pathway [15], [16]. More importantly, whereas previous studies [16], [24] characterized cancellation for a single stimulus strength, in this study we present the stimulus at different contrast levels (i.e. different strengths). We observe that the stimulus is cancelled efficiently for a wide range of input contrast levels, with cancellation values over 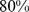 in all cases. Cancellation was measured by the ratio in gain between the local and global responses (see Methods for details).

Furthermore, the level of cancellation appears to be approximately the same for all contrasts, from very low (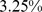) to very high (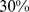) values (Fig. 1C), with a minor decay of cancellation levels observed for very high contrasts. Contrasts higher than 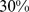 were not considered in this study, since P-unit electroreceptors encode AMs in a nearly linear manner up to 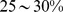. After that, the activity of P-units saturates and biologically relevant information can not be processed in the same quasi-linear regime [26]. When averaging over all the frequencies considered, we can observe that the degradation of the cancellation process (defined as the complementary of the frequency-averaged cancellation, i.e. 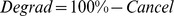) is restricted to a range between 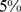 and 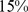, and therefore the cancellation of global signals only varies in about 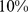 for all the range of biologically relevant contrast levels (Fig. 1D).

### Modeling local stimulation

We first consider the response of the SP neuron to local stimuli. The dynamics of the neuron membrane potential is modeled following a leaky integrate-and-fire (LIF) formalism [27], [28] with an extra burst-inducing mechanism. The subthreshold dynamics of the membrane potential is given by
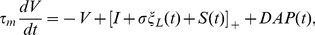
(1)where 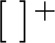 denotes rectification of the input, 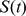 is the input from the P-units encoding the sensory stimulus, 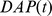 is the burst-inducing mechanism needed to reproduce the behavior of *in vivo* SP cells [29], [30], and 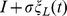 is a Gaussian low-pass filtered noise of mean 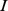 and standard deviation 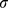 to fit the model to baseline (also referred here as *spontaneous*) activity conditions (i.e. no AM). As in the standard LIF formalism, when 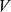 reaches a certain fixed threshold, a spike is recorded, and after that 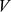 remains at a certain resting value during the absolute refractory period of the neuron.

**Figure 2:**
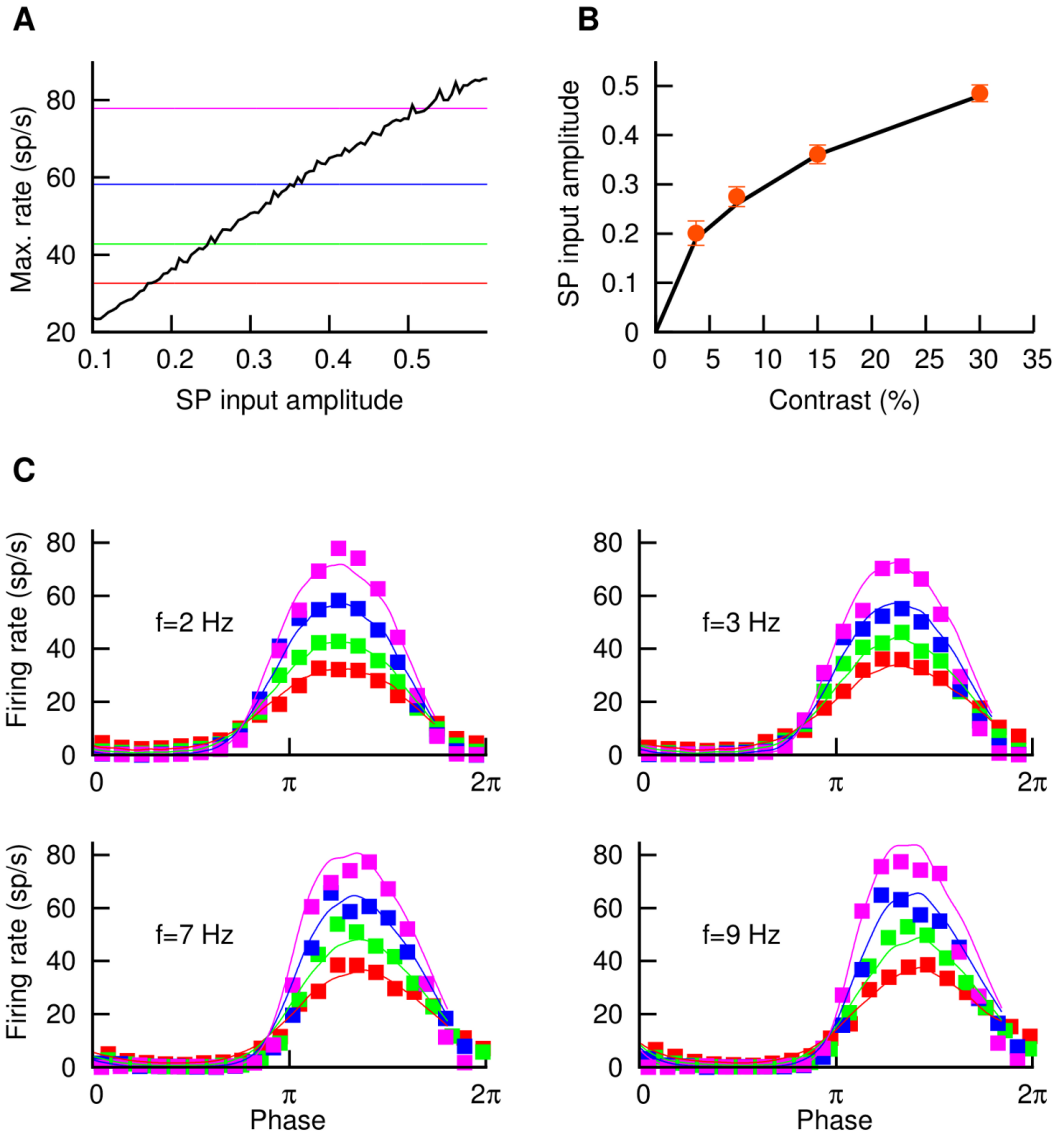
Fitting of the model to local response. (A) Maximum firing rate (solid black line) reached by the model SP neuron as a function of the amplitude of the sine wave 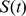 resembling the input from P-units. The AM frequency is 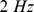, and similar responses were found for other frequencies. Colored lines indicate the maximum firing rate observed in the experiments for four different AM contrasts. (B) By considering the crossing points between the black line and the colored lines in panel A, we establish a dependence between AM contrasts (i.e. input to P-units) and amplitude of the signal arriving at the SP cell, 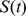, and thus obtaining an AM input-output function for the P-units. Points denote frequency-averaged quantities, while bars denote the standard deviation of each average. (C) Once this P-unit nonlinear feature is considered, the model (solid lines) is able to properly fit the experimental observations (points, shown previously in Fig. 1B) for different input frequencies and contrasts of the local stimulus. For panels A and C, the color code for contrast is red (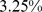 contrast), green (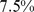), blue (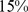) and violet (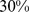).

When stimulated by a sinusoidal input, the model SP neuron responds with a modulation of its firing rate. Fig. 2A shows the maximum firing rate (solid black line) as a function of the amplitude of the sinusoidal-like signal entering the SP cell from the P-units. In color lines, we see the maximum firing rates observed experimentally for different contrasts entering the P-units. By looking at the intersections of the black curve with the color lines, we can determine the relationship between input contrast to the P-unit and input modulation to the SP cell (i.e. the P-unit output). The resulting input-output relationship for the P-units is shown in Fig. 2B. As we can see, the P-units display some degree of saturation for high input contrasts (of about 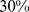). This agrees with previously known results [26] which show that P-units behave as linear encoders for relatively low contrasts (up to 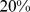), and beyond that point they start to saturate. We can now easily include such a saturation effect into our model (see section Methods). Once this nonlinearity has been considered, the response of our model agrees very well with the experimental observations for local stimuli, as one can see in Fig. 2C, for different AM frequencies and contrasts.

### Modeling global stimulation

We consider now the situation in which we have a spatially global stimulus in the system. The presence of the global stimulus activates the feedback pathway which projects onto the SP cells via the PFs. This implies considering an extra term in the dynamics of the membrane potential of the SP neuron, which is now
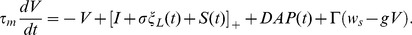
(2)In the last term, 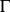 is the strength of the feedback (which will depend nonlinearly on the contrast since the feedback pathway is also driven by P-unit activity), and the term 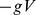 mimics the effect of disynaptic inhibition driven by the PFs. More precisely, since the reversal potential of the inhibitory synapse (GABA-A receptors) is close to the resting potential of the SP cell [31], inhibition was modelled as an extra shunting conductance [32]. The quantity 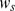 is the strength of the particular PF which is active (i.e. which is transmitting a burst arriving from a granule cell) at the phase segment 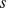 of the signal cycle. For simplicity, we assume that only one PF is active at a given time (see section Methods for details).

**Figure 3:**
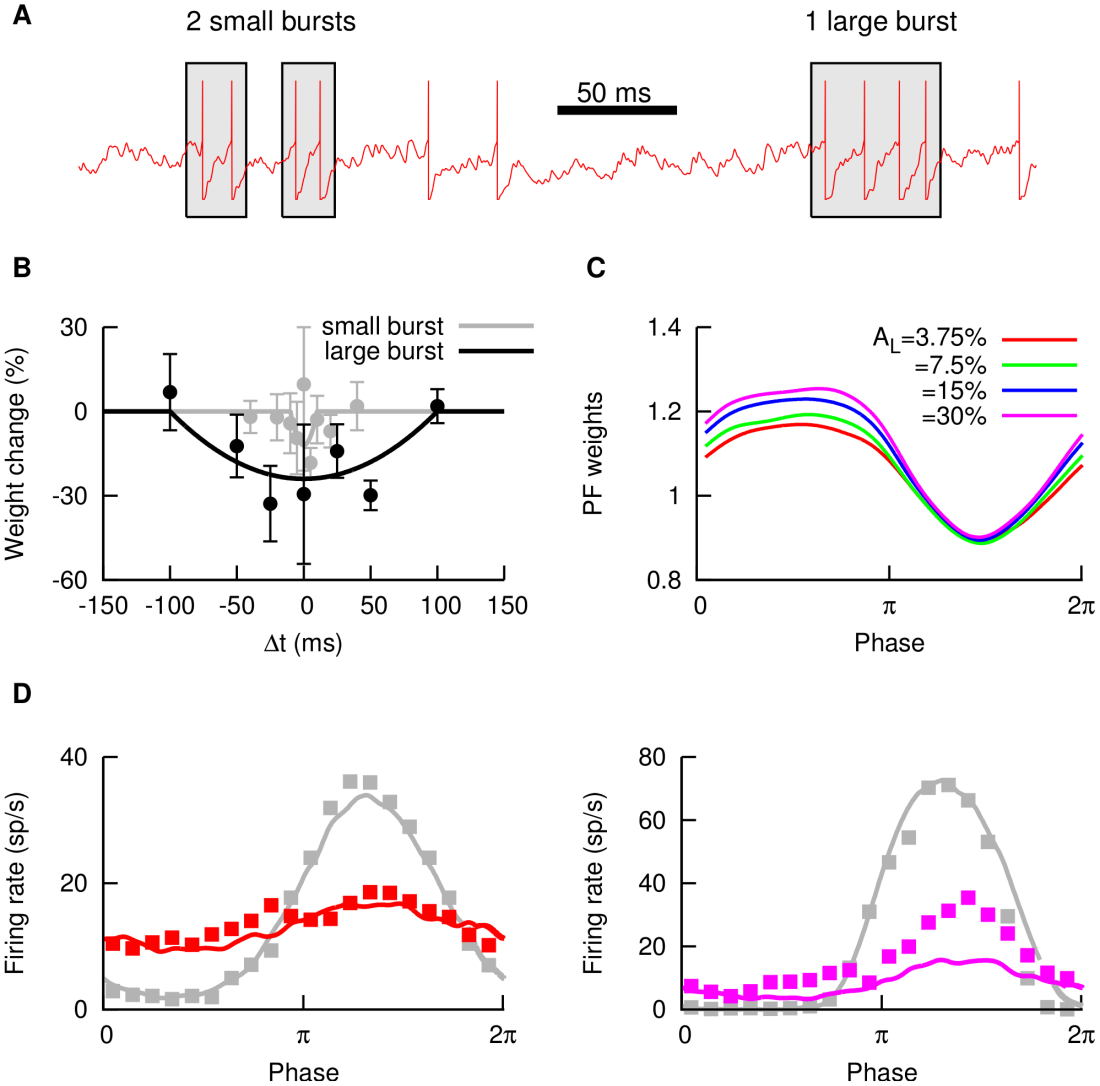
Parallel fiber weights and learning contrast. (A) Example of small and large bursts as defined in the text. Two spikes occurring within a 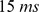 window constitute a small burst, whereas four spikes within 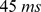 constitute a large burst. (B) Burst-driven learning rules for small and large bursts, as a function of the timing 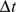 of the presynaptic and postsynaptic bursts. Points indicate data from *in vitro* recordings (taken from [23], each point being averaged over 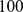 trials in the experimental plasticity protocol), and lines are the fit employed in the model. (C) PF weights as a function of the stimulus phase, for different learning contrasts 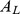. (D) Firing rate of the SP cell as a function of the stimulus phase, for local (gray) and global (colored) stimulation. The learning contrast was set at 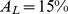 and the test contrast was 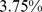 (left panel) or 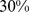 (right panel). For both panels, lines correspond to model predictions and points to experimental data shown previously in Fig. 1B.

At the SP cell, we distinguish between small and large bursts, since the characteristics of the learning rule will be different depending on the burst size [23]. Adopting the burst definitions which were explicitly characterized in [23], we will consider the 2-spike burst as the typical small burst, and the 4-spike burst as the typical large burst (see Fig. 3A, and section Methods for further details on burst definition). Small and large bursts have different roles in the cancellation process, as it was found in [16]: large bursts cover long periods associated with low-frequency input, while small bursts have a similar role for high frequencies and are also important in the timing of the plasticity for these input frequencies.

For burst timing purposes, the temporal location of a given burst is identified as the temporal location of its first spike. The burst-STDP learning rule employed, based on *in vitro* experimental recordings [23], is shown in Fig. 3B.

Every time a pair of presynaptic-postsynaptic bursts occur, each PF weight 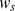 is updated according to the following rule
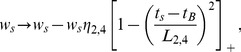
(3)where 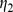 and 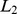 are used if the burst of the SP cell is a small burst, and 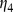 and 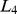 are used if it is a large burst. The presynaptic burst is assumed to match the burst type occurring at the SP cell. Once again, 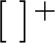 symbolizes rectification, which means this rule is applied to all weights whose phase segment began at a time 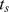 as long as 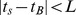. Beyond this range, the weights are unchanged.

Note that the burst-induced depression found *in vitro* is purely depressing and would eventually decrease all PF weights to zero. To avoid that, we include a non-associative potentiating rule so that the weights slowly relax back to 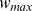 with a time constant of 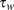 according to
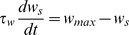
(4)This rule maintains the independence of synaptic weights and is biologically plausible, since Lewis and Maler [33] demonstrated a presynaptic form of synaptic enhancement in PFs. This enhancement was elicited when PF discharge occurred, without the need of a concomitant pyramidal cell burst response. Such a form of potentiation lasted for many minutes, and a weak potentiation with this or larger 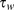 would have been difficult to detect experimentally. Furthermore, similar potentiation rules have been experimentally observed in mormyrid fish, and it has been shown to play an important role in cancellation in these fish [34], in which there is a corollary discharge.

Below we will see that the homeostatic time constant 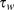 may play an interesting role in the learning dynamics. The response of the SP neuron model was always recorded after a certain *learning period*, during which all PF weights reached their equilibrium state.

One of the main points to take into account is that the strength of the PF synapses will depend on the stimulus contrast employed during the learning period (we denote such contrast level as *learning contrast*, 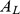). This is due to the fact that the occurrence of SP bursts, which are the driving events of PF plasticity, depends strongly on the stimulus contrast. The effect of the contrast on PF weights is shown in Fig. 3C, where one can see that the distribution of PF strengths is similar for different learning contrasts 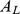, although not exactly the same. In all cases, the sinusoidal stimulus shapes the PF weights to form a negative image of the signal, which constitutes the basis of the cancellation phenomenon. The weights for different learning contrasts are almost identical around the peak of the stimulus (corresponding here to a phase of 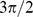), where the SP neuron is mainly driven by the stimulus (and noise plays a relatively minor role), and bursts are more likely to occur. The variability of PF weights with the learning contrast is higher for the signal trough (around phase of 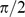), where bursts occur scarcely and are not able to efficiently shape the weight distribution. As we can see, PF weights around the signal trough are higher for high learning contrasts. This is due to the fact that a high-contrast stimulus induces a strong hyperpolarization in the SP membrane potential at the stimulus trough, lowering the chances of bursting for that stimulus phase and preventing the depressing LTP rule to decrease the weights.

Interestingly, even though both the feedforward and the feedback inputs are sinusoidally driven, the distribution of PF weights significantly deviates from a sinusoidal function, as one may clearly observe from Fig. 3C. Such a deviation has its origin in the highly nonlinear nature of burst dynamics in SP cells. Indeed, it is known that bursting rate displays a highly nonlinear, exponential-like relationship with input (see Fig. 5B in [24]). Since bursts are the main events driving the PF learning, the nonlinear input-burst relationship is translated into a nonlinearity in the PF weight distribution emerging from learning. This, successively, turns the feedback input to the SP cell into a highly nonlinear contribution that prevents treatment of the cancellation phenomenon as a trivial linear summation of sine waves that are out of phase with one another.

**Figure 5:**
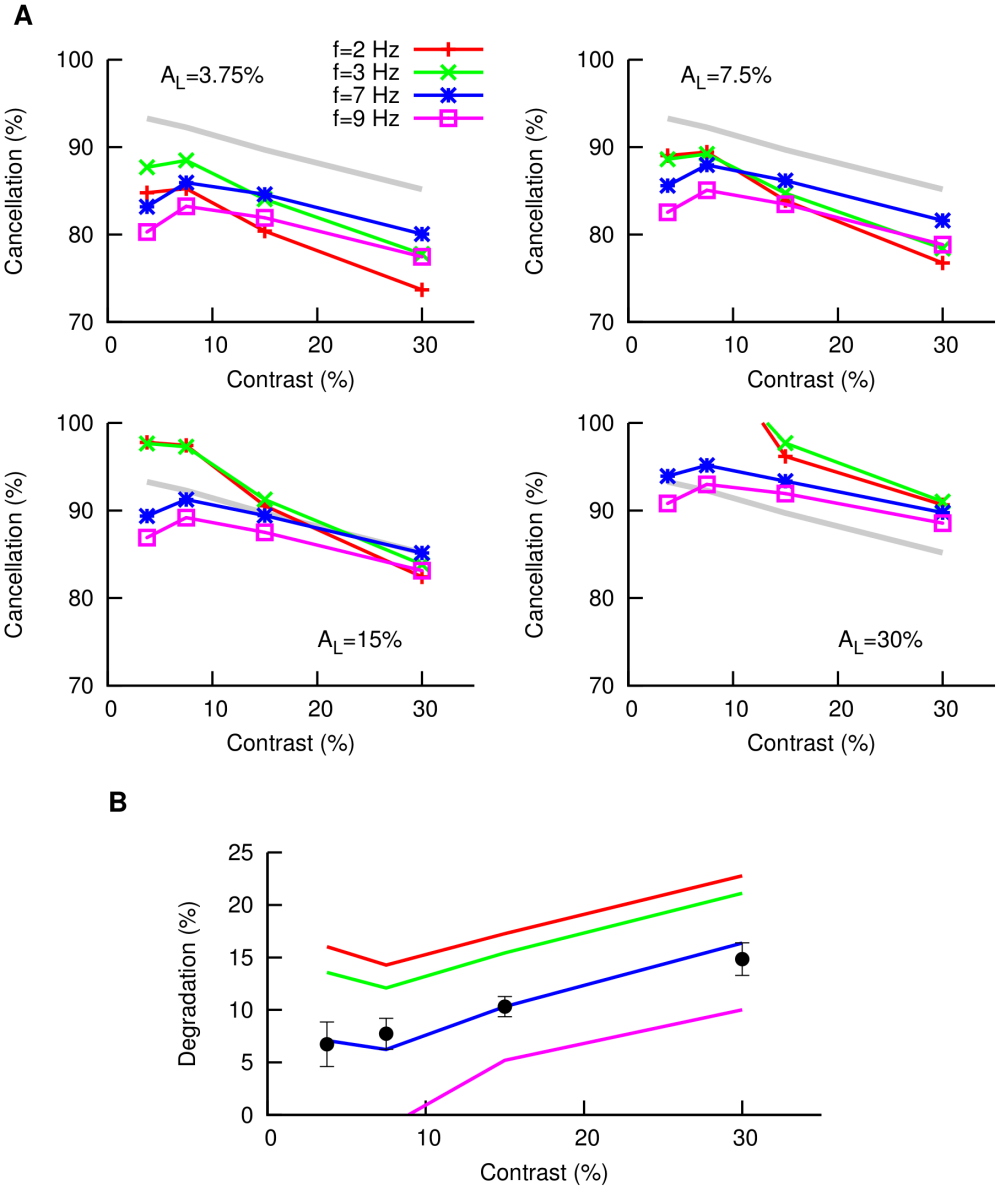
Cancellation of global stimulus with feedback saturation. (A) Model predictions of the level of cancellation of global signals as a function of contrast, for different signal AM frequencies (colored lines) and different learning contrasts considered (different panels). We have introduced here a factor 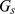 which takes into account the saturation of the feedback pathway for high stimulus contrasts. The gray line indicates the frequency-average cancellation levels measured experimentally (from data in Fig. 1C). (B) Degradation level (defined as the quantity 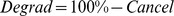, once frequency is averaged) as a function of the contrast for different learning contrasts (red 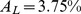, green 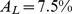, blue 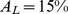, violet 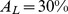). Symbols denote experimental data. As we can see, the optimal learning contrast is around 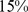.

In Fig. 3D, two examples of cancellation of a global signal of a frequency of 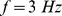, a learning contrast of 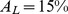, and different stimulus contrasts are shown (in each panel, the corresponding SP neuron response to same-frequency, same-contrast local signals is displayed in gray for comparison purposes). As we can see, the cancellation is very good in both cases, although the model overestimates the degree of cancellation for the 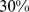 contrast case (violet line in right panel). In both panels, experimental data are plotted with points and model results are displayed with lines.

### Emergence of contrast invariance

The PF weights are modified via long-term plasticity mechanisms, which operate in the order of minutes to hours. Since changes in stimulus contrast associated with behavior (i.e. tail bending becoming narrower) may occur on the scale of milliseconds to seconds, one can not expect that the weights will be able to adapt fast enough to new presented contrasts in realistic situations. More likely, PF weights will reach some stationary level (as a result of some time-averaged contrast level provided by day-to-day natural stimuli), and then such an equilibrium level will be used to cancel any particular contrast level that the fish receives. In such a situation, we could expect certain differences in the quality of the cancellation depending on the learning contrast assumed in the simulations.

**Figure 4:**
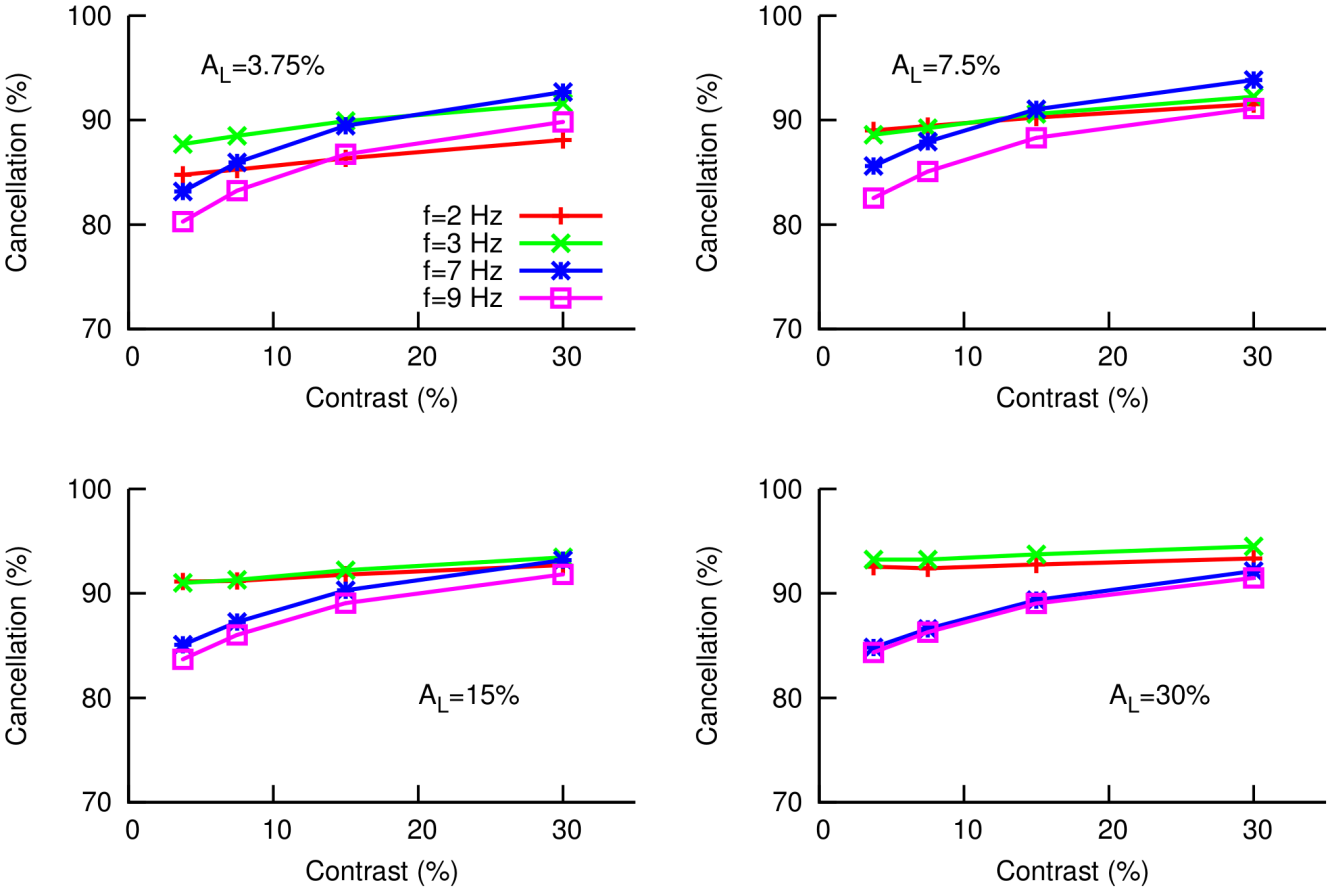
Cancellation of global stimulus without feedback saturation. Model predictions of the level of cancellation of global signals as a function of contrast, for different signal AM frequencies (colored lines) and different learning contrasts considered (different panels).

The model prediction of the cancellation level for different frequencies and contrasts, and assuming different learning contrasts, is shown in Fig. 4. In all cases, the cancellation levels are maintained on values over 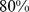 for different contrast levels, as in the experimental observations (see Fig. 1C), and thus indicate the emergence of contrast invariance in the cancellation of global stimuli in the model. It is particularly interesting to note, from the model results, that the specific learning contrast chosen has little impact on the results, contrary to what was expected, and that the levels of cancellation are broadly the same for all AM frequencies (with high frequencies having slightly lower values, as seen also in the experimental data).

Note that, in addition to this counterintuitive lack of impact of the learning contrast in the cancellation, some qualitative differences appear with respect to the experimental data. Concretely, for all frequencies and learning contrasts, the level of cancellation slightly increases with the stimulus contrast according to the model predictions, while in the experiments it slightly decreases. The origins of such model/experiment discrepancy may be diverse, but it is reasonable to assume that they could be mainly due to the lack of a key ingredient in the model's feedback pathway, since the response of the model and experiment for the local signal were in good agreement both qualitatively and quantitatively (see Fig. 2).

Since the discrepancy is mostly evident for large contrast values, one might want to consider, as a first approach, possible features of the real system that may be particularly relevant at those conditions. A relevant factor to consider here is the existence of saturation effects along the feedback pathway. Saturation is inherent to all spiking neurons, and high contrast input to granule cells might cause saturation in two, not mutually exclusive ways: higher contrasts might evoke discharge in a greater number of granule cells and/or it might evoke a higher frequency discharge in granule cells. The first situation was examined by Berman and Maler who used stronger PF stimulation to activate greater numbers of PFs; clear saturation of the PF response was observed (Fig. 5B in [32]). The second scenario was studied by Lewis and Maler [35]; this study demonstrated a saturating SP response to increasing PF stimulation frequency (Fig. 5 in [35]). The presence of saturation in the PF-SP synapses imposes a limit in the feedback strength for increasing stimulus contrasts, which would naturally lead to a devaluation of the cancellation quality for high contrasts as observed *in vivo*. Furthermore, it is reasonable to think that, in addition to this PF saturation, the bursting activity of granule cells might as well saturate for high enough contrast values (since only a limited number of granule cell bursts can be generated within one stimulus cycle), adding an extra layer of saturation to the feedback pathway.

To take into account this saturating behavior in the model, we consider a small correction in the feedback gain for high contrast values by introducing a factor 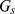 in the last term of Eq. 2. This extra factor will be one for low contrasts (i.e., 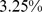 and 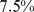) and less than one of higher values (we chose 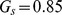 for 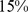 contrast and 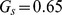 for 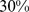 contrast, although other values are possible without qualitatively varying our conclusions). The cancellation levels with this new assumption are shown in Fig. 5A, for different frequencies and learning contrasts. As we can see now, the slight decrease in cancellation levels with increasing contrasts is present for all learning contrasts, in agreement with experimental data (shown as a gray line in panels of Fig. 5A for a direct visual comparison). Therefore, we have identified, via a computational model, the saturation of the feedback pathway as a plausible origin of the slight cancellation decrease with contrast. Our model also provides some insight into the plausible saturating dynamics of these granule cells, which have not been recorded *in vivo* to date.

### Optimal learning contrast

**Figure 6:**
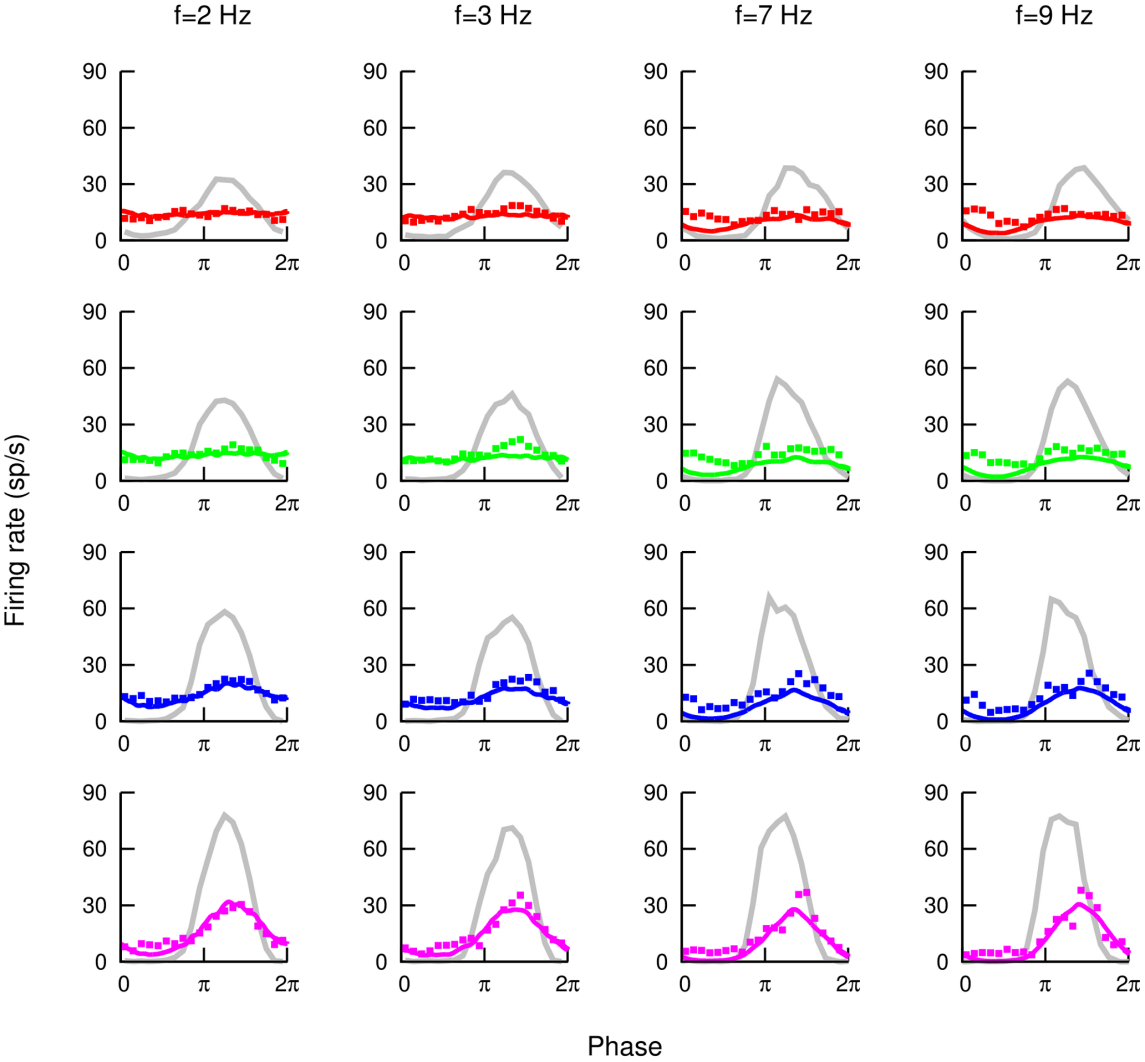
Cancellation at different frequencies and contrasts. Comparison between experimental data (points) and model predictions (lines) for the cancellation of a global signal for different AM frequencies (columns) and contrasts (red 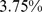 contrast, green 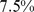 contrast, blue 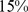 contrast, violet 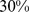 contrast). In each panel, the corresponding experimentally measured SP response to local stimulus is also shown in gray. The learning contrast chosen for the model was 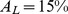, which optimizes the agreement between model predictions and experimental data.

A second conclusion that we can make from our modeling results concerns the level of cancellation for different learning contrasts. As we can see in Fig. 5A, considering different learning contrasts has now a clear effect on the cancellation properties, as opposed to the case in which saturation of the feedback pathway was not considered. Indeed, high learning contrasts shift the cancellation curves to higher values, leading to higher cancellation levels for all contrasts and frequencies considered. The closest agreement with the experimental data is obtained with a learning contrast of 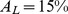, as we can see in Fig. 5A and more clearly in Fig. 5B. Lower learning contrasts lead to low values of overall cancellation, mainly because PF weights are tuned to cancel only weak modulations and are not able to overcome a large-amplitude signal completely. On the other hand, higher learning contrasts produce an over-cancellation at low input contrasts (that is, the SP cells have a peak of firing rate where the stimulus displays a trough, and vice versa) which is not observed experimentally. Due to the saturation of the feedback pathway with contrast, the PFs have to span a wider range of weight values in order to obtain a proper cancellation, and this has a negative effect when trying to cancel low contrast signals. Therefore, the optimal learning contrasts are those situated just below the appearance of a strong saturation in the feedback pathway, but strong enough to allow cancellation for the whole regime of linear encoding of P-units, around 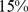. For such an optimal learning contrast, individual firing rate responses are also shown in Fig. 6 as a function of the global stimulus phase for different contrasts and frequencies. The figure also shows the corresponding experimental SP response to local stimulation for comparison purposes.

**Figure 7:**
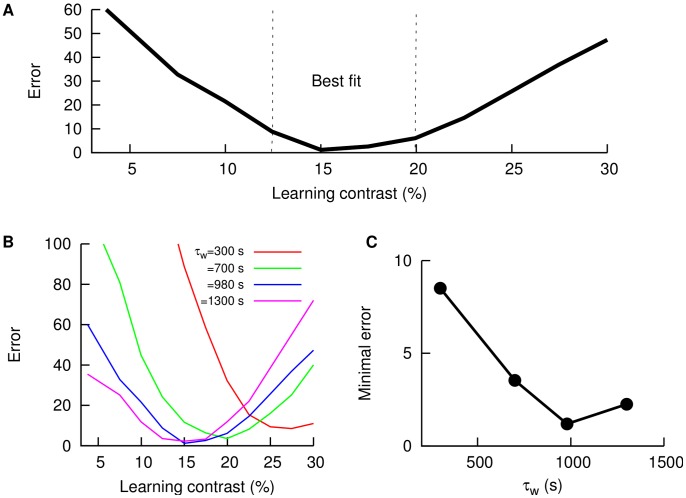
Optimal learning contrast and potentiation time constant. (A) Error function, defined as the sum of the squared distances between experimental and model points, as a function of the learning contrast. Dashed lines, located at 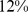 and 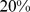, enclose the region of learning contrast levels which give a reasonably good fit (Error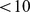) between data and simulations. (B) Same as panel A, but for different time constant values of the potentiation learning rule for comparison. (C) Minimum of each error curved displayed in panel B, as a function of the potentiation time constant. The lowest error is obtained for potentiation time constants of about 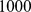 seconds.

It might be argued that 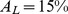 is only optimal when compared to the few other values of the learning contrast considered here. To better characterize the optimal learning contrast and its robustness, we have employed our model to extend our study and to consider other learning contrasts. We consider now a range of possible learning contrasts (around eleven values from 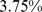 to 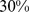) and we compute, for each one of them, the degradation of cancellation as a function of the stimulus contrast, as in Fig. 5B. For learning contrasts not considered in the experiments, such as 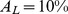, values for the P-unit adaptation and feedback saturation in the model were obtained by linear interpolation between known values. We also define a function which quantifies the discrepancy between the model prediction for a given 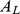 and the experimental data. The error function is given by
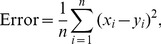
(5)where 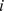 runs over all stimulus contrasts considered (up to 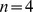 for an easier comparison with experimental data), and 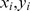 are, respectively, the model and experimental degradation for the stimulus contrast 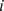. As we can see in Fig. 7A, the error function is minimal for 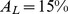. Considering surrounding contrast levels with similar error function values would give us a range of optimal learning contrast of 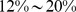 contrast levels, which correspond to reasonably low error levels in the figure. Interestingly, contrast levels around this range are commonly found within the natural environment of the weakly electric fish (Yu *et al.*, personal communication). In particular, it has been experimentally shown that the presence of free-swimming conspecifics induces a certain range of contrast levels in the electric fish, being these levels centered and more common around 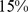 (see Fig. 3B in [25]), in strong agreement with the optimal contrast level predicted by our model.

In addition to identifying an optimal learning contrast around 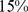, our model gives us insight into the dynamics of weak PF potentiation. As it has been argued, the potentiation rule is hard to find experimentally, since it is expected to work at very long time scales and would therefore have a hardly appreciable effect during *in vitro* recordings [23] (associative potentiation rules have been, however, found in mormyrid fish [34]). To study the potentiation time scale in detail, we evaluate the error measurement defined above as a function of the learning contrast, for different values of the potentiation time constant 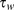. As Fig. 7B shows, different 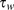 values lead to different error curves. Time constants of 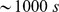 or above yield mainly the same results, i.e. the optimal learning contrast lies around 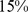. Smaller time constants significantly deviate from this value, which is to be expected since a small time constant would lead to more rapid forgetting of the phase-specific synaptic strength and thus significantly modify the learning rule and the PF weight distributions. The model learning rule would thus not fit the experimental data anymore. For time constants of 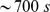 or lower, the optimal learning contrast is found to be higher, which makes intuitive sense because the system forgets faster, but the minimal error reached in these cases is substantially larger than for larger time constants. This can be clearly seen in Fig. 7C: the minimal error decreases as 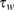 increases, until a global minimum is reached for 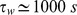 (corresponding to an optimal learning contrast of about 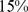). This value for the potentiation time constant is therefore a strong prediction of our model. *In vitro* experiments that pharmacologically eliminate the confounding effects of postsynaptic depression [23] and disynaptic inhibition [35] should be able to precisely estimate 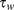 and therefore test our prediction.

## Discussion

Removal of redundant information is a key task to accomplish for an optimal detection of novel stimuli in sensory systems. Unfortunately, not many neural mechanisms are known to provide such a filtering under realistic conditions. In this work, we have analyzed one of the few neural circuits clearly identified as a system able to cancel redundant information, which involves the indirect feedback pathway to the ELL of the weakly electric fish *Apteronotus leptorhynchus*. Our results, obtained from a combination of *in vivo* extracellular recordings and detailed computational modeling, reveal a plausible framework which explains the cancellation of redundant information observed in the electric fish [15], [16]. They further reveal that this cancellation displays contrast invariance over the entire range of behaviorally relevant contrast levels [26]. The key ingredients for this contrast-invariant cancellation can be summarized in (*i*) the efficient transmission of the contrast level through the (nonlinear) feedback pathway, resulting in a match of the feedback input to the feedforward signal in the SP, and (*ii*) the fact that, when the PFs adjust their synaptic weights to cancel a given contrast, they provide a good cancellation input for other contrasts as well. Due to these two features, the model is able to explain the high levels of cancellation (over 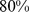 at all times) observed in experiments for a broad range of AM frequencies and contrasts, thus providing a theoretical framework for the contrast invariance in cancellation. This theoretical framework may be helpful to understand other neural systems where cancellation of redundant signals occurs (such as auditory systems [36] or other neural circuits confronting “cocktail party” problems [1], [2]) and may also provide novel useful points of view to understand contrast invariance in visual systems [6], [9]. In addition, our finding might be seen as a very simple form of context-specific adaptation [37], since the adaptation mechanism (the feedback input into SP cells) would make the SP response different depending on the behavioral context (e.g. prey vs conspecific).

In order to achieve the good agreement of this model with the experimental data, an additional ingredient has been necessary to explain the minor decay of cancellation levels with contrast found *in vivo*. According to our modeling results, a weak saturation in the feedback pathway is a sufficient condition to explain the decay in cancellation for high stimulus contrasts, and this saturation may have different sources. It is known, for instance, that a strong PF stimulation activating a large number of PFs induces a prominent saturation in the PF transmission [32]. Such a saturation phenomenon in PFs would be enough to provide the weak level of saturation needed to explain our experimental findings. Furthermore, PFs are also known to saturate for increasing frequency [35], and other factors such as a possible saturation in granule cell firing or the presence of short-term plasticity mechanisms found in PF synapses [38] may also contribute to the saturation of the feedback pathway. In particular, short-term synaptic depression could be a plausible candidate to induce feedback saturation, as it can induce synaptic fatigue causing nonlinear gain control [39] and, when interacting with short-term facilitation, can produce important effects in the dynamics of recurrent neural circuits [35], [40], [41].

While we have assumed in this work that granule cells in the EGp fire in a bursty fashion and phase-lock to the periodic stimulus, there is an ongoing debate concerning the propensity of the granule cells to fire in bursts [42]–[46]. Importantly, we are dealing here with a specialized group of cerebellar cells, the Zebrin-2 negative cells [47], whose firing patterns are not known to date. However, the presence of granule cells with a strong bursting behavior is not required for our conclusions to hold. Since the global stimulus is of a periodic nature, it will likely induce the clustering of granule cell spikes around a certain range of stimulus phases, even if the granule cells do not have a tendency to burst. A simple scaling of the granule cell response with the stimulus, as it occurs for SP cells, is therefore the only essential requirement of our model. Similarly, the concrete input/output characteristics of granule cells do not have a strong impact on our results, as saturation for large contrasts has already been found experimentally in parallel fibers [32], and therefore it is not necessary to impose this condition to granule cells.

The possible role of different types of inhibition in the cancellation of global signals has been experimentally addressed previously. For instance, Maler *et al.* [48] presented morphological evidence suggesting the presence of lateral inhibition (but not recurrent inhibition), as the one we are considering in our model. Bastian *et al.* [49] demonstrated the existence of inhibitory surrounds for superficial pyramidal cells, but it was later observed [15] that the cancellation could be completely prevented by blocking the EGp feedback, and thus suggesting that these inhibitory surrounds do not have an important role for cancellation. Therefore, the role of other types of inhibition can not be completely ruled out, but their effects on cancellation have been found to be much less important than the PF-triggered feedback inhibition that we are considering in our study. On the other hand, local input activates feedforward inhibition, but this type of input does not trigger cancellation as we also illustrate in Fig. 1B.

It is also important to mention that, due to the fact that the same stimulus is driving both the SP cells (via the feedforward pathway) and the EGp (via the feedback pathway), parallel fibers will be naturally time-locked to the stimulus. As a consequence, any initial phase displacement in the stimulus (with respect to previous stimuli) will affect both the SP cells and the EGp in the same way and will not affect the cancellation. Sudden and fast phase shifts like the ones associated with communication signals (i.e. small chirps [50]), however, will not be predicted and cancelled by the present mechanism, and they will be treated as novel stimuli since they may carry useful behavioral information.

In addition to different stimulus contrasts, we have considered signals with different AM frequencies in our study. Local signals (such as prey), which should not be cancelled, usually fall into the range of frequencies considered here (from 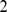 to 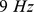). Such a range of frequencies lies within the band of good cancellation observed experimentally and, consequently, we do not observe major differences among AM frequencies in the cancellation level [16]. A slight decrement in cancellation is however observed for 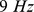, which is indeed to be expected since cancellation starts to decay around 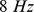 and is practically inexistent at 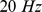 [16], [24]. On the other hand, while frequency-specific channels have been identified in this system [16], the width of a given frequency channel is not known to date. In our model, we have assumed that frequencies as close as 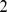 and 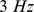 are canceled via different frequency-specific channels, but it might be possible that both frequencies fall into the cancellation domain of a single channel. However, the detailed mechanisms that such a broadband channel could employ to cancel close (but different) frequencies are unknown and they fall out of the scope of our study. Therefore we assumed here that each frequency was processed by a specific channel. The good agreement between our experimental observations and model predictions suggests that our approach may be indeed adequate.

It is also worth mentioning that the specific definition of burst does not have a fundamental importance in our model. In particular, the one used here (i.e. the occurrence of a number of spikes within a fixed time window) has been chosen for being computationally adequate, but also for being consistent from a biophysical point of view. This is explained by the following two factors: (*i*) the ISI distribution of SP cells is bimodal [16], with a clear frequency-independent peak at small ISI values which highlights the existence of bursting [51], and (*ii*) in our system, plasticity is not triggered by single-pulse paired stimulation [23]. The combination of these two factors suggests that SP bursts are structured and well located in time, and highlights the importance of bursts as functionally meaningful events which clearly differ from single spikes.

The PF plasticity rule, as presented in [23], would constantly weaken PF strength until all synapses would reach zero strength. To avoid that, a weak, phase-independent potentiation mechanism was considered here following previous studies [16]. This potentiation rule might have to be extremely slow such that its effect was not detected in standard *in vitro* protocols. Such a plasticity mechanism would therefore be hard to measure for most direct methods. Aided by our model and experimental findings, we were able to predict a reasonable value for the potentiation time constant, of about 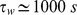. This estimation constitutes a strong prediction of our model, and further modeling and experimental studies will be necessary to corroborate and extend this prediction.

Our model indicates the existence of a certain *optimal learning contrast*, which presumably resembles the time-averaged contrast that should drive the PF plasticity to obtain the cancellation values observed experimentally. We have found that this optimal learning contrast lies around 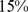 contrast levels (or, considering a small range of contrast levels, around 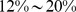). Interestingly, contrast levels around this value are commonly found within the natural environment for communication signals in the weakly electric fish, such as, for instance, when surrounded by free-swimming conspecifics [25]. Indeed, assuming that a global AM of 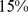 contrast level is due to the presence of a conspecific, this would correspond roughly to a distance of 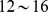 cm between both fish [52]. In addition, this indicates that a close experimental measurement of the common contrast levels found in the fish's natural environment (which has been the goal of recent studies [25]) might provide a good indirect confirmation of the existence of weak potentiation rules which are hard to find in *in vitro* conditions. Further experimental and modeling work is needed, however, to clarify these possible links, as well as the impact of other realistic assumptions in our circuit, such as considering heterogeneous populations of superficial neurons [53], [54].

Finally, the study of cancellation of global signals needs to be extended to situations in which more realistic stimuli are considered. Although of remarkable usefulness, the assumption of global sinusoidal signals would correspond only to the case of a perfectly periodic tail movement, or to the presence of a static conspecific at a certain fixed distance. However, tail bending is commonly an aperiodic movement in real conditions and, in addition, the distance between two electric fish would constantly vary as they swim. This implies that the stimulus contrast will vary in time, as for instance following an Ornstein-Uhlenbeck process as shown by Yu *et al.* [25], and such variations are likely to be relevant in the cancellation process. This constitutes a much more complex situation than the one studied here (in which each of the contrast levels we have investigated is constant in time), although preliminary work suggests that the present framework may be extended to explain the cancellation for those complex situations as well. For instance, in a complex global signal constituted by several coexisting frequency components, each one of them could be cancelled independently by frequency-specific channels present in the feedback circuit [16]. Furthermore, slow variations in the contrast level for any given frequency might activate other adaptation mechanisms that could aid in the cancellation, as we are currently investigating.

## Methods

### Ethics statement

All experimental procedures were approved by the University of Ottawa Animal Care Committee.

### *In vivo* electrophysiology

Experimental recordings were performed as in [16]. Briefly, craniotomy is performed under general anesthesia. During the experiment the fish is awake but paralyzed with curare and locally anesthetized. Single-unit extracellular recordings from superficial pyramidal E cells of the centro-lateral segment of the electrosensory lateral line lobe were performed. These cells can be easily identified based to their location (depth and centro-medial position), their receptive field, their baseline firing rate and response properties. Stimuli consisted of amplitude modulations of the fish's own electric field. The stimulus was delivered through two large global electrodes placed on each side of the fish thereby achieving a global stimulation. For local stimulation, a small dipole was placed in the center of the cell's receptive field; the distance between the dipole and the skin was adjusted to maximally stimulate the whole receptive field of the cell while avoiding stimulation of receptors outside the classical receptive field.

### Network architecture

**Figure 8:**
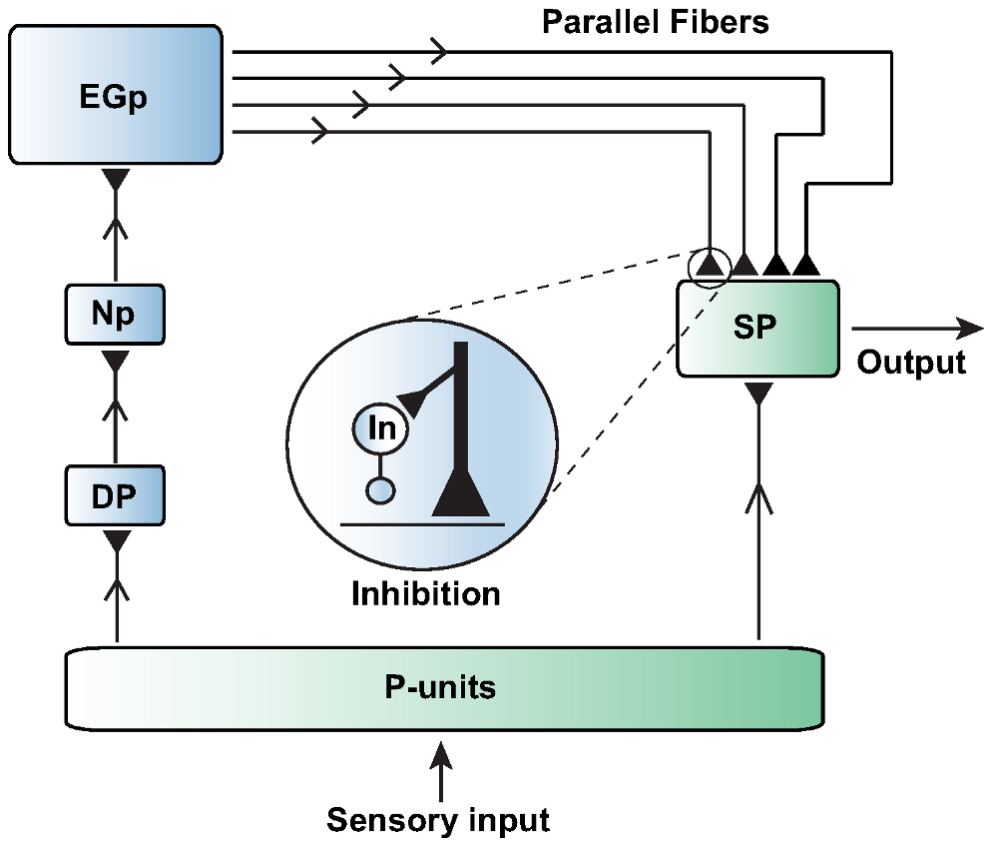
Schematic diagram of the model considered. The network architecture considered in the model involves the feedforward circuit (in green) and the indirect feedback pathway (in blue), which is active only for global stimulus.

To understand the biological mechanisms responsible for contrast-invariant cancellation of global stimuli, we consider a simplified model of the ELL and the indirect feedback pathway (see Fig. 8). AMs of the sensory input are encoded in firing rate modulations of the P-units. If the stimulus is spatially local, P-units transmit these modulations to the SP neuron, which projects to other higher brain regions. If the stimulus is spatially global, the feedback pathway is also activated (in addition to the feedforward pathway sketched above) and a population of EEL neurons called deep pyramidal (DP) cells transmit the signal from the P-units to a granule cell population, the eminential granularis posterior (EGp), via the Nucleus praeminentialis (nP). Each granule cell projects through parallel fibers onto the SP neuron, closing the feedback loop.

In the real system, the cerebellar feedback pathway to the ELL is bilateral [18], [55] and can take several routes before returning to the ELL. Furthermore, DP cells, which constitute the origin of the feedback pathway, display a variety of phase relationships with the stimulus depending on its location (i.e., the side of the body) and the specific cell type (E-cells, which fire at the signal peak, or I-cells, firing at the trough) [56], [57]. Finally, each granule cell will be located at a certain position in space and therefore it will be characterized by a particular distance to the target SP cell. All these elements together produce a wide range of feedback temporal delays, suggesting that the PFs can provide feedback to SP neurons at all possible phases of the global periodic stimulus. In addition to this, granule cells have been reported to phase-lock to periodic signals and to burst to sensory stimuli and be silent elsewhere [43], [45], [58], [59].

To include these features in our simplified model, we assume that (1) the array of PFs provide feedback to SP neurons at all possible phases of the sensory stimulus, and (2) granule cells in the feedback pathway respond in a bursty fashion, phase-locked to the AM frequency signal. We also take into account in the model that PFs also synapse onto inhibitory interneurons, which provide some level of inhibition to the SP neuron.

It has also been shown that certain long-term plasticity rules may adjust the weights of the parallel fibers. According to recent *in vitro* experiments, PFs projecting onto SP cells display a long-term depression (LTD) rule that depends on the timing of presynaptic and postsynaptic bursts [23]. Such a burst-driven learning rule, combined with the presence of PFs displaying a wide set of temporal delays, is thought to be responsible for the generation of a negative image of the input AMs, providing the substrate for signal cancellation [16].

### LIF model

For local stimulation, the SP neuron is modelled following a leaky integrate-and-fire (LIF) formalism (Eq. 1) with an extra term accounting for the bursting dynamics (DAP). Noise was introduced in the system via a low-pass filtered (with cut-off frequency 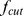) Gaussian noise 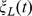 of zero mean and variance one. The variance is later adjusted via the parameter 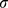, and a constant bias 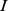 is introduced to fit the experimental firing rate in spontaneous (i.e. no AMs) conditions. As in the standard LIF model, a spike is recorded when 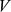 reaches the threshold 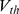, and after that 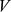 remains at a certain resting value 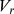 during the absolute refractory period 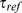 of the neuron.

The EOD signal arriving at electroreceptors can be described, as a first approach, as a sinewave of amplitude 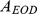 and high frequency (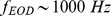). The presence of stimuli induces EOD amplitude modulations of frequency 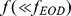 and contrast 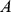, so that the EOD amplitude is also a sinewave given by 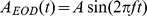. Since electroreceptors encode AMs by modulating their firing rate, the AM frequency 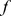 and the contrast 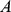 are enough to characterize their behavior. The output firing rate 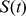 of the P-unit population, which is driven by this input, is given by
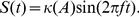
(6)P-units are known to display some level of saturation for high contrasts [26] (this effect is denoted in the above equation as 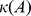), and we observe such saturation in our *in vivo* recordings via the nonlinear response of the SP cell to different stimulus contrasts. By fitting the model SP cell response to the *in vivo* measured SP cell response, we determine the input-output amplitude relationship of the P-units (see Fig. 2A and B). The corresponding values are 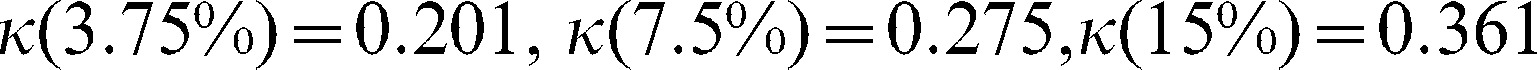 and 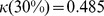. When using values other than these ones, linear interpolation was employed to estimate the new values of 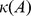. To incorporate the effect of P-unit adaptation at low frequencies (as in [16]), we multiply the signal 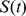 by a small constant factor of 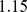 for 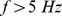.

In addition, as electroreceptor input is strictly excitatory, the input to the SP neuron is rectified and 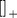 in Eq. 1 symbolizes rectification (that is, 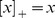 if 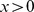, and 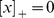 otherwise). This aided us in incorporating the rectified nature of the pyramidal cell activity into the model. Values for these parameters are displayed in Table 1.

**Table 1:** Parameter values for the equations of the model which approximate experimental data.

Parameter	Value
*V_th_*	1
*V_r_*	0
*τ_m_*	7 *ms*
*τ_ref_*	0.7 *ms*
*I*	0.59
*σ*	0.768
*g*	1.44
*f_cut_*	500 *Hz*
*τ_w_*	980 *s*
*w_max_*	1.5
*η*_2_	0.0018
*η*_4_	0.0036
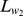	10 *ms*
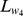	100 *ms*

### Bursting mechanism

Superficial cell bursting drives the long-term plasticity rules operating in the PFs of the feedback pathway. The term 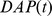 in Eq. 1 models the depolarizing after-potential (DAP), an injection of current into the soma of the neuron after an action potential is fired due to presence of active channels in the cell's dendrites. This effect has been modeled previously in superficial cells [29], [30], and we adapt the parameter values used in these works to match the bursting rate of the model to our experimental observations.

The mechanism responsible for the generation of bursting is the following: after the cell fires (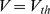) at time 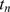, it will receive a DAP (i.e. a small current injection) a short time later, as long as the previous time the cell fired is not too recent. This additional stimulation is modeled as a difference in alpha functions 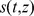 (Eq. 8), one generated by the soma voltage, and the other by some mean dendrite voltage. However, if the time interval between this spike time 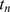 and the previous spike time 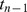 is less than the refractory period of the dendrite, 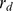, then the DAP is inactive for the current spike. The refractory period 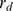 is modeled as a dynamic variable 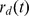 that changes according to a secondary variable, 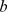, which is updated for each spike. The time just after the most recent spike was fired is referred as 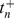. The equations governing the DAP [30] are
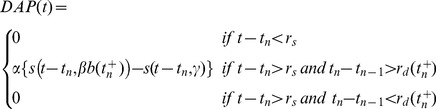
(7)

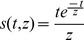
(8)

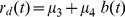
(9)

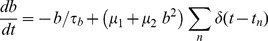
(10)The parameters used in the above equations are listed in Table 2.

**Table 2:** Parameters used in the DAP model.

Parameter	Value
*α*	20
*β*	2.45 *ms*
*γ*	1.4 *ms*
*μ*_1_	0.6
*μ*_2_	2
*μ*_3_	0.7 *ms*
*μ*_4_	24.5 *ms*
*r_s_*	0.7 *ms*
*τ_b_*	7 *ms*

### Parallel fibers

The feedback pathway is initiated by DP cells, which do not exhibit global cancellation behavior since they do not receive feedback, and these cells project onto the nucleus praeminentialis (nP) which in turn projects onto EGp granule cells. Finally, granule cells project massive numbers of excitatory PFs to the ELL, where they provide input to SP cells as well as local interneurons (stellate cells). The stellate cells in turn inhibit the SP cells via shunting GABA-A receptor channels.

Due to difficulties in recording them, the firing activity of EGp granule cells in the electric fish is not known. Similar granule cells in mammals, however, have been shown to respond to sensory input [43], [58] and to phase-lock their bursting to sinusoidal input [45]. We assume here, therefore, that the activity of each PF is one burst per stimulus period.

Considering the natural distribution of temporal PF delays (see details on network architecture above), the total bursting PF input was assumed to be continuous in time. In the model, the feedback cycle associated with the stimulus cycle was discretized into segments of 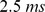 each. This implies that the number of segments changes with the AM frequency considered (for instance, we would have 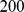 feedback segments for a 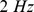 stimulus, and 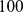 segments for an 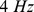 stimulus). Each segment, labeled 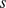, becomes active at time 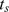, has a global strength 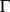 (common for all PFs) and a synaptic weight 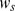 (particular for each PF, see Eq. 2), and then becomes inactive at 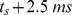. Each segment is associated with the activity of a given PF for simplicity, although it could be associated with the activity of a certain set of coincident PFs as well. The total excitatory feedback input is therefore a step-wise continuous and periodic signal given by 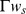, for each segment 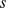 as time moves from segment to segment during a period. Disynaptic inhibition, which is also modulated by PF activity, is modeled as an extra shunting conductance 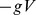.

The global strength of the feedback is driven by DP cells, which in turn are driven by P-units. This implies a dependence of the feedback strength on the P-unit response which is modeled as a dependence of 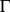 with the contrast 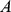:
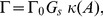
(11)with 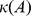 being the input/output relationship of the P-units. The parameter 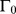 is set to 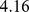 for the global stimulus (in order to fit the mean firing rate measured experimentally at global stimulation of 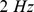 and 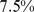 contrast), and to zero for the local stimulus (since this type of stimulus does not activate the feedback pathway). The saturation of the feedback pathway is being taken into account in the parameter 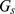, which will be one for low contrasts (i.e., 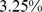 and 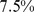) and less than one of higher values (we chose 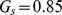 for 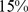 contrast and 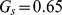 for 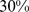 contrast). When feedback saturation is not being considered in the model, we just set 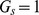 for all contrasts. Again, since P-units drive feedback, the P-unit adaptation at low AM frequencies discussed above will affect as well the feedback input. Therefore, we follow the same criterion as with 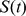 and we multiply the whole feedback function by a small factor 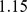 if 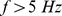 to account for P-unit adaptation.

A necessary condition for cancellation is to have a stable phase relationship for each segment and, hence, each weight. Such a requirement is fulfilled by considering that there is a particular set of PFs responsible for the cancellation of a given AM frequency. This has been corroborated with *in vivo* electrophysiological measurements [16], and therefore we assume here that our feedback pathway is frequency-specific.

As also observed experimentally in [16], cancellation starts to decay at high AM frequencies, hypothetically due to failures of granule cells for bursting at least once per cycle under global stimulation (and thus failing to drive learning properly). In agreement with these observations, we notice a slightly consistent decay of cancellation for 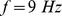, which can be easily taken into account by reducing 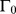 to a value of 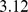 for this frequency, to improve the fitting of experimental data.

### Burst definition and cancellation measurement

Following the definition of a burst that induces plasticity [23], the spike train of the model SP cell was constantly monitored for small (2 spikes within 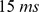) and large (4 spikes within 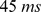) bursts. These particular definitions of burst are only adopted here to simplify the online computations, as the quantitative behavior of our model does not depend sensitively on such assumptions, or even on the presence of strong intrinsic mechanisms for bursting generation (see Discussion for details).

It is worth mentioning here that spikes in each SP burst must be independent (i.e. there cannot be a small burst in a large burst, or a large and a small burst in 5 spikes). Since each PF segment produces a presynaptic burst arriving at the apical dendrite, there is one PF burst at every time 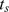 in the model, and thus PF bursts are spaced 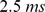 apart. When the SP cell bursts under global stimulation at time 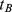, the burst learning rule identified *in vitro* (Eq. 3) is immediately invoked for all PF segments.

To quantify the level of cancellation of the global stimuli, we follow [16] and employ the following criterion:
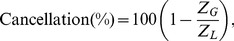
(12)where 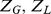 are, respectively, the amplitude of SP response (measured in spikes per second) for global and local stimulation. More precisely, the PSTH (i.e. firing rate as a function of stimulus phase) of the SP cell was fitted to a sinusoidal function (plus baseline level) for the global stimulation case, and the amplitude of such sinusoidal was taken as 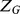. Because of the rectification, the same fit could not be applied to the local stimulation case, since the response clearly deviates from a sine wave. The SP response to local stimulation was then fitted to a Gaussian distribution (plus baseline level) and the height of such a Gaussian was taken as 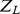. This criterion was followed for both experimental data and model predictions.

The degradation measurement is the complementary of the frequency-averaged cancellation, and it was employed to show, in a clear manner, how much the cancellation level is degraded when increasing the stimulus contrast (Fig. 1D). It is defined as
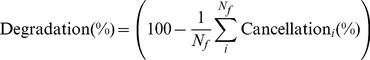
(13)with 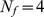 is the number of AM frequencies considered in the study (which are 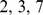 and 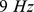) and the sum runs over all these frequencies.
